# Supplementary material for: Increased heterogeneity and task-related reconfiguration of functional connectivity during a lexicosemantic task in autism
Source: Neuroimage Clin. 2024 Oct 28;44:103694. doi: 10.1016/j.nicl.2024.103694 (PMC11574795; doi:10.1016/j.nicl.2024.103694)
Supplement: Supplementary Data 1 [file mmc1.docx]

**Supplemental Materials**

Supplemental Table S1. Psychotropic Medication Use in the ASD Group

| **Participant** | **Stimulants** | **Mood Stabilizers^a^** | **SSRI/**  **Antidepressants** | **Anxiolytics/ Other^b^** | **List of medications** |
| --- | --- | --- | --- | --- | --- |
| **a** | **+** | **+** | **+** | **+** | Mirtazapine, Escitalopram oxalate, Aripiprazole, Methylphenidate hydrochloride |
| **b** | **+** |  |  | **+** | Methylphenidate hydrochloride, Guanfacine |
| **c** | **+** |  |  |  | Methylphenidate hydrochloride |
| **d** | **+** |  |  |  | Methylphenidate Hydrochloride |
| **e** | **+** | **+** | **+** | **+** | Oxcarbazepine, Guanfacine, Aripiprazole, Escitalopram oxalate, Alprazolam |
| **f** |  |  |  | **+** | Inderal |
| **g** |  | **+** |  |  | Aripiprazole |
| **h** |  | **+** | **+** | **+** | Guanfacine, Aripiprazole, Bupropion Hydrochloride |
| **i** | **+** | **+** | **+** |  | Aripiprazole, Lisdexamfetamine, Dextroamphetamine, Fluoxetine, Citalopram |
| **j** |  | **+** | **+** | **+** | Quetiapine, Sertraline, Duloxetine, Amantadine, Prazosin |
| **k** |  | **+** | **+** |  | Clomipramine, Sertaline, Bupropion, Divalproex |
| **Total** | **7** | **7** | **6** | **6** |  |

Supplemental Table S2. Word Category Statistics

|  | | **Frequency** | **Age of Acquisition (AoA)** | **Number of letters** | **Number of syllables** |
| --- | --- | --- | --- | --- | --- |
| **SW** | Mean (SD) | 3.8 (0.7) | 6.8 (1.8) | 6 (2.0) | 1.9 (0.9) |
|  | Range | 2.3 – 5.6 | 3.3 -11.6 | 3 -11 | 1 -5 |
| **AW** | Mean (SD) | 3.4 (0.7) | 6.4 (1.7) | 5.9 (1.7) | 2.0 (0.7) |
|  | Range | 1.6 – 4.9 | 3.6 -10.6 | 2 -9 | 1-4 |
| **PW** | Mean (SD) | -- | -- | 6.1 (2.1) | 2.1 (1.0) |
|  | Range | -- | -- | 2-11 | 1-5 |
| **SW vs AW** | *t* (df) =  *p*-values | 4.07 (238) < 0.001 | 1.43 (238) = 0.16 | 0.62 (238) = 0.53 | -0.67 (238) = 0.51 |
| **AW vs PW** | *t* (df) =  *p*-values | -- | -- | -0.14 (118) = 0.89 | -1.06 (118) = 0.29 |
| **SW vs PW** | *t* (df) =  *p*-values) | -- | -- | 0.09 (238) = 0.93 | -1.36 (238) = 0.17 |

Supplemental Table S3. Peak Coordinates of Regions of Interest

|  | **X** | **Y** | **Z** | **Voxels** |
| --- | --- | --- | --- | --- |
| **L/R middle cingulate cortex** | 9.5 | 44.5 | 30.5 | 91 |
| **L inferior & superior parietal lobules** | 24.5 | 68.5 | 33.5 | 51 |
| **L/R anterior cingulate cortex** | 6.5 | -36.5 | -11.5 | 75 |
| **L middle occipital gyrus** | 42.5 | 77.5 | 21.5 | 76 |
| **L inferior parietal lobule** | 54.5 | 59.5 | 21.5 | 84 |
| **R angular gyrus** | -32.5 | 62.5 | 39.5 | 41 |
| **R middle occipital gyrus** | -44.5 | 77.5 | 18.5 | 77 |
| **L middle temporal gyrus** | 57.5 | 32.5 | 0.5 | 64 |
| **R Rolandic operculum** | -53.5 | 17.5 | 9.5 | 66 |
| **L postcentral gyrus** | 57.5 | 20.5 | 12.5 | 53 |
| **L calcarine gyrus** | 12.5 | 59.5 | 12.5 | 54 |
| **R precentral gyrus** | -38.5 | 26.5 | 45.5 | 89 |
| **L supplemental motor area** | 6.5 | -15.5 | 36.5 | 80 |
| **L precentral gyrus** | 42.5 | 2.5 | 36.5 | 78 |
| **L inferior frontal gyrus** | 39.5 | -21.5 | 21.5 | 20 |
| **L superior temporal gyrus** | 54.5 | -12.5 | -11.5 | 88 |

Supplemental Table S4. Subgroup Demographics

|  | **TD_s_ (n = 19)** | | **LP-ASD (n =15)** | | **TP-ASD (n = 15)** | |  |
| --- | --- | --- | --- | --- | --- | --- | --- |
| **Gender** | 6 Female | | 4 Female | | 5 Female | |  |
| **Handedness** | 2 left | | 0 left | | 1 left | |  |
|  | Mean (SD) | Range | Mean (SD) | Range | Mean (SD) | Range |  |
| **Age in years** | 15.8 (2.3) | 12.1 - 21.0 | 15.33 (2.6) | 10.0 - 20.0 | 15.67 (2.7) | 12.1 – 20.0 |  |
| **RMSD** | 0.06 (0.02) | 0.03 - 0.12 | 0.08 (0.02) | 0.04 - 0.11 | 0.07 (0.02) | 0.03 – 0.09 |  |
| **WASI-II** |  |  |  |  |  |  |  |
| Nonverbal IQ | 112.0 (9.9) | 90 - 128 | 106.1 (22.4) | 62 -156 | 108.1 (21.6) | 62 - 132 |  |
| Verbal IQ | 113.1 (12.8) | 85 - 135 | 98.4 (17.8) | 68 -124 | 110.4 (16.7) | 68 - 134 |  |
| Full Scale | 114.9 (12.0) | 93 - 135 | 100.3 (19.6) | 54 -136 | 111.1 (21.7) | 54 - 141 |  |
| **WIAT-III** | 111.2 (8.5) | 100 - 129 | 93.3 (19.0) | 59 -128 | 109.0 (18.0) | 59 - 133 |  |
| **ADOS-2 Total**^✝^ | -- | -- | 10.9 (3.7) | 6 - 20 | 10.6 (3.8) | 2 - 16 |  |
| **BRIEF-2 GEC** | 45.0 (7.7) | 36 - 62 | 66.5 (5.8) | 56 - 74 | 65.9 (9.6) | 49 - 84 |  |
| **CELF-5 WC** | 36.2 (2.2) | 32 - 39 | 34.0 (3.8) | 27 -39 | 35.9 (4.4) | 27 - 40 |  |
| **Psychotropic medication use** | -- | | 6 reported | | 5 reported |  |  |

Supplemental Table S5. Subgroup Demographic Statistics

|  | **LP-ASD vs TD_s_** | **TP-ASD vs TD_s_** | **LP-ASD vs TP-ASD** | |
| --- | --- | --- | --- | --- |
|  | *t* (df), *p* - value | | |  |
| **Age in years** | 0.4 (32), *p* = 0.69 | 0.8 (32), *p* = 0.41 | -0.4 (28), *p* = 0.73 |  |
| **RMSD** | 2.2 (32), *p* = 0.03 | 1.3 (32), *p* = 0.21 | -1.2 (28), *p* = 0.25 |  |
| **WASI-II** |  |  |  |  |
| Nonverbal IQ | -1.0 (32), *p* = 0.31 | -0.7 (32), *p* = 0.49 | -0.2 (28), *p* = 0.81 |  |
| Verbal IQ | -2.8 (32), ***p* = 0.01 | -0.5 (32), *p* = 0.60 | -1.9 (28), *p* = 0.07 |  |
| Full Scale | -2.7 (32), **p* = 0.01 | -0.7 (32), *p* = 0.52 | -1.4 (28), *p* = 0.16 |  |
| **WIAT-III** | -3.7 (32), ***p* = 0.001 | -0.5 (32), *p* = 0.64 | -2.3 (28), **p* = 0.03 |  |
| **ADOS-2 Total**^✝^ | -- | -- | 0.2 (28), *p* = 0.81 |  |
| **BRIEF-2 GEC** | 8.7 (30), ****p* < 0.001 | 6.3 (26), ****p* < 0.001 | 0.2 (22), *p* = 0.85 |  |
| **CELF-5 WC** | -2.2 (32), **p* = 0.04 | -0.3 (32), *p* = 0.78 | -1.3 (28), *p* = 0.21 |  |

*** *p*<.001, ** *p*<.01 * *p*<.05.

Supplemental Table S6. Task Performance per Sample and Between Sample Comparisons

|  |  | **ASD** | | **TD** | | ***t* (df), *p*-value** |
| --- | --- | --- | --- | --- | --- | --- |
|  |  | **Mean (SE)** | **Range** | **Mean (SE)** | **Range** |  |
| **SW** | **Accuracy** | 0.90 (0.02) | 0.67 – 1.0 | 0.97 (0.01) | 0.90 – 0.99 | -3.1(51), ***p* = 0.003 |
|  | **RT** | 946.4 (25.7) | 630.2 – 1227.4 | 837.9 (28.7) | 582.0 – 1106.0 | 2.1 (51), **p* = 0.04 |
| **AW** | **Accuracy** | 0.80 (0.03) | 0.45 – 1.0 | 0.90 (0.01) | 0.77 – 0.97 | -2.5 (51), **p* = 0.02 |
|  | **RT** | 938.7 (20.9) | 679.0 – 1148.8 | 850.7 (26.6) | 601.3 – 1137.1 | 1.9 (51), *p* = 0.06 |
| **PW** | **Accuracy** | 0.91 (0.02) | 0.67 – 1.0 | 0.97 (0.02) | 0.68 – 1.0 | -1.6 (51), *p* = 0.11 |

*** p<.001, ** p<.01 * p<.05.

Supplemental Table S7. Task Performance and Comparisons Between Conditions per Group

|  |  | **ASD**  ***t* (df), *p*-value** | **TD**  ***t* (df), *p*-value** |
| --- | --- | --- | --- |
| **SW vs AW** | **Accuracy** | *t* (29) = 4.4, ****p* < 0.001 | *t* (22) = 7.0, ****p* < 0.001 |
|  | **RT** | *t* (29) = 0.8, *p* = 0.45 | *t* (22) = -1.0, *p* = 0.31 |
| **AW vs PW** | **Accuracy** | *t* (29) = -4.4, ****p* < 0.001 | *t* (22) = -4.0, ****p* < 0.001 |
| **PW vs SW** | **Accuracy** | *t* (29) = -0.3, *p* = 0.73 | *t* (22) = -0.9, *p* = 0.38 |

*** *p*<.001, ** *p*<.01 * *p*<.05.

Supplemental Table S8. FDR-corrected *p-* values for Group Differences in Functional Connectivity

| **Connectivity (ROI pairing)** | **t- statistic (df) = *p* - values** | | |
| --- | --- | --- | --- |
|  | **Rest** | **Task** | **Reconfiguration** |
| L/R middle cingulate cortex-L inferior & superior parietal lobules | 0.52(51) = 0.28 | 0.98(51) = 0.33 | 0.74(51) = 0.46 |
| L/R middle cingulate cortex-L/R anterior cingulate cortex | 0.92(51) = 0.46 | 1.19(51) = 0.24 | 1.43(51) = 0.16 |
| L/R middle cingulate cortex-L middle occipital gyrus | 0.39(51) = 0.25 | 1.32(51) = 0.19 | 0.94(51) = 0.35 |
| L/R middle cingulate cortex-L inferior parietal lobule | 0.96(51) = 0.97 | 2.01(51) = 0.05 | 1.13(51) = 0.26 |
| L/R middle cingulate cortex-R angular gyrus | 0.87(51) = 0.72 | 0.89(51) = 0.38 | 0.67(51) = 0.5 |
| L/R middle cingulate cortex-R angular gyrus | 0.35(51) = 0.12 | 0.64(51) = 0.52 | 1.05(51) = 0.3 |
| L/R middle cingulate cortex-L middle temporal gyrus | 2.28(51) = 0.56 | 2.31(51) = 0.03 | -0.18(51) = 0.86 |
| L/R middle cingulate cortex-R Rolandic operculum | 1.47(51) = 0.13 | 1.51(51) = 0.14 | 0.35(51) = 0.73 |
| L/R middle cingulate cortex-L postcentral gyrus | 0.1(51) = 0.57 | 0.41(51) = 0.68 | 1.53(51) = 0.13 |
| L/R middle cingulate cortex-L calcarine gyrus | 1.69(51) = 0.06 | 1.96(51) = 0.06 | 0.36(51) = 0.72 |
| L/R middle cingulate cortex-R precentral gyrus | 0.14(51) = 0.56 | 0.13(51) = 0.89 | 2.02(51) = 0.05 |
| L/R middle cingulate cortex-L SMA, L middle cingulate cortex | 1.23(51) = 0.55 | 1.44(51) = 0.15 | -0.01(51) = 0.99 |
| L/R middle cingulate cortex-L precentral gyrus | 0.97(51) = 0.92 | 1.33(51) = 0.19 | 0.29(51) = 0.77 |
| L/R middle cingulate cortex-L inferior frontal gyrus | 0.32(51) = 0.31 | 0.82(51) = 0.42 | 0.38(51) = 0.71 |
| L/R middle cingulate cortex-L superior temporal gyrus | 0.89(51) = 0.74 | 1.06(51) = 0.29 | 0.73(51) = 0.47 |
| L inferior & superior parietal lobules-L/R anterior cingulate cortex | 0.38(51) = 0.61 | 0.69(51) = 0.49 | 1.46(51) = 0.15 |
| L inferior & superior parietal lobules-L middle occipital gyrus | 0.51(51) = 0.8 | 1.11(51) = 0.27 | 2.46(51) = 0.02 |
| L inferior & superior parietal lobules-L inferior parietal lobule | 1.16(51) = 0.42 | 0.91(51) = 0.37 | 1.39(51) = 0.17 |
| L inferior & superior parietal lobules-R angular gyrus | 0.83(51) = 0.99 | 1.2(51) = 0.24 | 1.89(51) = 0.06 |
| L inferior & superior parietal lobules-R angular gyrus | -0.36(51) = 0.83 | 0.58(51) = 0.56 | 1.99(51) = 0.05 |
| L inferior & superior parietal lobules-L middle temporal gyrus | 1.15(51) = 0.99 | 0.54(51) = 0.59 | 0.74(51) = 0.46 |
| L inferior & superior parietal lobules-R Rolandic operculum | 1.32(51) = 0.89 | 1.19(51) = 0.24 | 1.78(51) = 0.08 |
| L inferior & superior parietal lobules-L postcentral gyrus | 1.56(51) = 0.48 | 1.52(51) = 0.13 | 1.44(51) = 0.16 |
| L inferior & superior parietal lobules-L calcarine gyrus | 0.5(51) = 0.45 | 0.53(51) = 0.6 | 1(51) = 0.32 |
| L inferior & superior parietal lobules-R precentral gyrus | 0.93(51) = 0.97 | 1.41(51) = 0.16 | 1.04(51) = 0.3 |
| L inferior & superior parietal lobules-L SMA, L middle cingulate cortex | 0.6(51) = 0.79 | 0.56(51) = 0.57 | 2.75(51) = 0.01 |
| L inferior & superior parietal lobules-L precentral gyrus | 1.19(51) = 0.71 | 0.42(51) = 0.67 | 1.77(51) = 0.08 |
| L inferior & superior parietal lobules-L inferior frontal gyrus | 1.04(51) = 0.83 | 1.45(51) = 0.15 | 2.72(51) = 0.01 |
| L inferior & superior parietal lobules-L superior temporal gyrus | 1.43(51) = 0.81 | 1.05(51) = 0.3 | 2.22(51) = 0.03 |
| L/R anterior cingulate cortex-L middle occipital gyrus | -0.2(51) = 0.51 | 0.08(51) = 0.94 | 2.03(51) = 0.05 |
| L/R anterior cingulate cortex-L inferior parietal lobule | 1.7(51) = 0.33 | 1.94(51) = 0.06 | 1.86(51) = 0.07 |
| L/R anterior cingulate cortex-R angular gyrus | 1.57(51) = 0.92 | 1.76(51) = 0.08 | 1.69(51) = 0.1 |
| L/R anterior cingulate cortex-R angular gyrus | 2.25(51) = 0.41 | 2.3(51) = 0.03 | 0.68(51) = 0.5 |
| L/R anterior cingulate cortex-L middle temporal gyrus | 1.25(51) = 0.53 | 1.99(51) = 0.05 | 0.78(51) = 0.44 |
| L/R anterior cingulate cortex-R Rolandic operculum | 2.33(51) = 0.1 | 2.62(51) = 0.01 | 0.79(51) = 0.43 |
| L/R anterior cingulate cortex-L postcentral gyrus | 0.5(51) = 0.17 | 0.69(51) = 0.49 | 1.41(51) = 0.17 |
| L/R anterior cingulate cortex-L calcarine gyrus | -0.11(51) = 0.9 | -0.11(51) = 0.92 | 1.96(51) = 0.06 |
| L/R anterior cingulate cortex-R precentral gyrus | 1.14(51) = 0.13 | 0.86(51) = 0.39 | 1.74(51) = 0.09 |
| L/R anterior cingulate cortex-L SMA, L middle cingulate cortex | 1.05(51) = 0.25 | 1.84(51) = 0.07 | 1.33(51) = 0.19 |
| L/R anterior cingulate cortex-L precentral gyrus | 0.67(51) = 0.82 | 1.35(51) = 0.18 | 1.61(51) = 0.11 |
| L/R anterior cingulate cortex-L inferior frontal gyrus | 0.62(51) = 0.46 | 0.49(51) = 0.63 | 1.02(51) = 0.31 |
| L/R anterior cingulate cortex-L superior temporal gyrus | 0.86(51) = 0.17 | 1.49(51) = 0.14 | 1.01(51) = 0.32 |
| L middle occipital gyrus-L inferior parietal lobule | 0.96(51) = 0.88 | 0.49(51) = 0.63 | 0.4(51) = 0.69 |
| L middle occipital gyrus-R angular gyrus | 1.24(51) = 0.85 | 1.55(51) = 0.13 | 1.34(51) = 0.19 |
| L middle occipital gyrus-R angular gyrus | -0.61(51) = 0.11 | 0.15(51) = 0.88 | 0.32(51) = 0.75 |
| L middle occipital gyrus-L middle temporal gyrus | 1.83(51) = 0.25 | 2.51(51) = 0.02 | 0.62(51) = 0.54 |
| L middle occipital gyrus-R Rolandic operculum | 0.76(51) = 0.76 | 1.17(51) = 0.25 | 1.13(51) = 0.27 |
| L middle occipital gyrus-L postcentral gyrus | -0.33(51) = 0.46 | 0.08(51) = 0.94 | 1.61(51) = 0.11 |
| L middle occipital gyrus-L calcarine gyrus | 0.02(51) = 0.92 | 0.73(51) = 0.47 | -0.77(51) = 0.44 |
| L middle occipital gyrus-R precentral gyrus | 0.28(51) = 0.89 | 0.35(51) = 0.72 | 1.81(51) = 0.08 |
| L middle occipital gyrus-L SMA, L middle cingulate cortex | 0.63(51) = 0.74 | 1.47(51) = 0.15 | 0.82(51) = 0.42 |
| L middle occipital gyrus-L precentral gyrus | 1.14(51) = 0.2 | 1.87(51) = 0.07 | 1.83(51) = 0.07 |
| L middle occipital gyrus-L inferior frontal gyrus | 0.19(51) = 0.67 | 0.59(51) = 0.56 | -0.81(51) = 0.42 |
| L middle occipital gyrus-L superior temporal gyrus | 0.58(51) = 0.59 | 1.03(51) = 0.31 | 1.97(51) = 0.05 |
| L inferior parietal lobule-R angular gyrus | 1.34(51) = 0.87 | 1.04(51) = 0.31 | 1.81(51) = 0.08 |
| L inferior parietal lobule-R angular gyrus | 0.81(51) = 0.99 | 1.04(51) = 0.3 | 2.58(51) = 0.01 |
| L inferior parietal lobule-L middle temporal gyrus | 1.46(51) = 0.5 | 1.45(51) = 0.15 | 0.69(51) = 0.5 |
| L inferior parietal lobule-R Rolandic operculum | 1.09(51) = 0.57 | 1.46(51) = 0.15 | 1.06(51) = 0.29 |
| L inferior parietal lobule-L postcentral gyrus | 0.31(51) = 0.69 | 0.46(51) = 0.65 | 1.57(51) = 0.12 |
| L inferior parietal lobule-L calcarine gyrus | 1.59(51) = 0.94 | 2.17(51) = 0.03 | -0.74(51) = 0.46 |
| L inferior parietal lobule-R precentral gyrus | 0.46(51) = 0.87 | 0.68(51) = 0.5 | -0.14(51) = 0.89 |
| L inferior parietal lobule-L SMA, L middle cingulate cortex | 0.31(51) = 0.82 | 0.29(51) = 0.77 | 0.99(51) = 0.33 |
| L inferior parietal lobule-L precentral gyrus | 1.17(51) = 0.63 | 1.28(51) = 0.21 | 1.76(51) = 0.08 |
| L inferior parietal lobule-L inferior frontal gyrus | -0.01(51) = 0.55 | -0.29(51) = 0.77 | 1.02(51) = 0.31 |
| L inferior parietal lobule-L superior temporal gyrus | 0.68(51) = 0.75 | 0.89(51) = 0.38 | 1.08(51) = 0.28 |
| R angular gyrus-R angular gyrus | 1.09(51) = 0.89 | 1.22(51) = 0.23 | 2.57(51) = 0.01 |
| R angular gyrus-L middle temporal gyrus | 1.84(51) = 0.55 | 1.27(51) = 0.21 | 1.83(51) = 0.07 |
| R angular gyrus-R Rolandic operculum | 1.32(51) = 0.79 | 1.3(51) = 0.2 | 2.13(51) = 0.04 |
| R angular gyrus-L postcentral gyrus | 1.27(51) = 0.95 | 1.13(51) = 0.26 | 1.08(51) = 0.29 |
| R angular gyrus-L calcarine gyrus | 0.53(51) = 0.46 | 0.92(51) = 0.36 | 1.2(51) = 0.24 |
| R angular gyrus-R precentral gyrus | 0.77(51) = 0.8 | 1.04(51) = 0.31 | 1.35(51) = 0.18 |
| R angular gyrus-L SMA, L middle cingulate cortex | 0.63(51) = 0.3 | 0.67(51) = 0.5 | 1.81(51) = 0.08 |
| R angular gyrus-L precentral gyrus | 1.62(51) = 0.34 | 1.24(51) = 0.22 | 1.7(51) = 0.1 |
| R angular gyrus-L inferior frontal gyrus | 1.12(51) = 0.96 | 1.53(51) = 0.13 | 0.97(51) = 0.34 |
| R angular gyrus-L superior temporal gyrus | 0.93(51) = 0.79 | 0.79(51) = 0.43 | 2.41(51) = 0.02 |
| R angular gyrus-L middle temporal gyrus | 3(51) = 0.34 | 2.92(51) = 0.01 | 1.07(51) = 0.29 |
| R angular gyrus-R Rolandic operculum | 1.65(51) = 0.58 | 1.9(51) = 0.06 | 2.63(51) = 0.01 |
| R angular gyrus-L postcentral gyrus | 0.63(51) = 0.47 | 0.9(51) = 0.37 | 2.71(51) = 0.01 |
| R angular gyrus-L calcarine gyrus | -0.43(51) = 0.45 | 0.15(51) = 0.88 | -0.45(51) = 0.65 |
| R angular gyrus-R precentral gyrus | 0.47(51) = 0.7 | 0.47(51) = 0.64 | 2.7(51) = 0.01 |
| R angular gyrus-L SMA, L middle cingulate cortex | 1.06(51) = 0.94 | 1.39(51) = 0.17 | 1.33(51) = 0.19 |
| R angular gyrus-L precentral gyrus | 1.07(51) = 0.88 | 1.16(51) = 0.25 | 3.09(51) = 0 |
| R angular gyrus-L inferior frontal gyrus | 0.7(51) = 0.82 | 0.96(51) = 0.34 | 2.13(51) = 0.04 |
| R angular gyrus-L superior temporal gyrus | 1.26(51) = 0.63 | 1.23(51) = 0.22 | 2.17(51) = 0.03 |
| L middle temporal gyrus-R Rolandic operculum | 1.83(51) = 0.66 | 1.73(51) = 0.09 | 2.23(51) = 0.03 |
| L middle temporal gyrus-L postcentral gyrus | 1.39(51) = 0.82 | 1.52(51) = 0.14 | 0.37(51) = 0.71 |
| L middle temporal gyrus-L calcarine gyrus | 2.04(51) = 0.31 | 2.34(51) = 0.02 | -0.53(51) = 0.6 |
| L middle temporal gyrus-R precentral gyrus | 0.83(51) = 0.69 | 1.09(51) = 0.28 | 0.36(51) = 0.72 |
| L middle temporal gyrus-L SMA, L middle cingulate cortex | 0.09(51) = 0.75 | -0.42(51) = 0.68 | 0.09(51) = 0.93 |
| L middle temporal gyrus-L precentral gyrus | 0.26(51) = 0.44 | -0.23(51) = 0.82 | 1.87(51) = 0.07 |
| L middle temporal gyrus-L inferior frontal gyrus | 0.16(51) = 0.52 | 0.06(51) = 0.95 | 0.52(51) = 0.61 |
| L middle temporal gyrus-L superior temporal gyrus | 0.31(51) = 0.54 | 0.05(51) = 0.96 | 0.42(51) = 0.68 |
| R Rolandic operculum-L postcentral gyrus | 2(51) = 0.56 | 1.38(51) = 0.17 | 1.19(51) = 0.24 |
| R Rolandic operculum-L calcarine gyrus | 1.04(51) = 0.06 | 1(51) = 0.32 | 0.71(51) = 0.48 |
| R Rolandic operculum-R precentral gyrus | 0.71(51) = 0.84 | 0.58(51) = 0.57 | 1.37(51) = 0.18 |
| R Rolandic operculum-L SMA, L middle cingulate cortex | 2.41(51) = 0.29 | 2.25(51) = 0.03 | 0.02(51) = 0.99 |
| R Rolandic operculum-L precentral gyrus | 1.5(51) = 0.66 | 1.48(51) = 0.14 | 3.37(51) = 0 |
| R Rolandic operculum-L inferior frontal gyrus | 1.72(51) = 0.94 | 1.97(51) = 0.05 | 1.21(51) = 0.23 |
| R Rolandic operculum-L superior temporal gyrus | 2.08(51) = 0.39 | 2.08(51) = 0.04 | 1.27(51) = 0.21 |
| L postcentral gyrus-L calcarine gyrus | -0.22(51) = 0.31 | -0.53(51) = 0.6 | 1.18(51) = 0.25 |
| L postcentral gyrus-R precentral gyrus | 2.1(51) = 0.83 | 1.48(51) = 0.14 | 0.73(51) = 0.47 |
| L postcentral gyrus-L SMA, L middle cingulate cortex | 1.97(51) = 0.44 | 2.11(51) = 0.04 | 0.27(51) = 0.79 |
| L postcentral gyrus-L precentral gyrus | 1.8(51) = 0.94 | 1.64(51) = 0.11 | 2.06(51) = 0.04 |
| L postcentral gyrus-L inferior frontal gyrus | 1.67(51) = 0.46 | 1.92(51) = 0.06 | 0.89(51) = 0.38 |
| L postcentral gyrus-L superior temporal gyrus | 2.04(51) = 0.53 | 2.29(51) = 0.03 | -0.02(51) = 0.99 |
| L calcarine gyrus-R precentral gyrus | 0.11(51) = 0.34 | -0.32(51) = 0.75 | -0.1(51) = 0.92 |
| L calcarine gyrus-L SMA, L middle cingulate cortex | 1.09(51) = 0.41 | 1.46(51) = 0.15 | -0.06(51) = 0.95 |
| L calcarine gyrus-L precentral gyrus | 0.38(51) = 0.59 | 0.98(51) = 0.33 | 1.49(51) = 0.14 |
| L calcarine gyrus-L inferior frontal gyrus | 0.06(51) = 0.19 | 0.78(51) = 0.44 | 1.31(51) = 0.2 |
| L calcarine gyrus-L superior temporal gyrus | 1(51) = 0.32 | 1.18(51) = 0.24 | 0.94(51) = 0.35 |
| R precentral gyrus -L SMA, L middle cingulate cortex | 1.09(51) = 0.58 | 1.15(51) = 0.26 | 0.52(51) = 0.6 |
| R precentral gyrus -L precentral gyrus | 0.53(51) = 0.16 | 0.99(51) = 0.33 | 0.5(51) = 0.62 |
| R precentral gyrus -L inferior frontal gyrus | 1.35(51) = 0.89 | 1.52(51) = 0.13 | 0.88(51) = 0.38 |
| R precentral gyrus -L superior temporal gyrus | 1.6(51) = 0.7 | 1.72(51) = 0.09 | 0.66(51) = 0.51 |
| L SMA, L middle cingulate cortex-L precentral gyrus | 0.93(51) = 0.3 | -0.07(51) = 0.94 | 1.29(51) = 0.2 |
| L SMA, L middle cingulate cortex-L inferior frontal gyrus | 0.45(51) = 0.83 | 0.65(51) = 0.52 | 2.67(51) = 0.01 |
| L SMA, L middle cingulate cortex-L superior temporal gyrus | 1.36(51) = 0.87 | 0.59(51) = 0.56 | -0.27(51) = 0.79 |
| L precentral gyrus-L inferior frontal gyrus | 1.82(51) = 0.81 | 1.94(51) = 0.06 | 0.28(51) = 0.78 |
| L precentral gyrus-L superior temporal gyrus | 1.32(51) = 0.92 | 0.67(51) = 0.51 | 1.2(51) = 0.23 |
| L inferior frontal gyrus-L superior temporal gyrus | 0.95(51) = 0.91 | 1.26(51) = 0.21 | 1.27(51) = 0.21 |

All p-values are uncorrected.


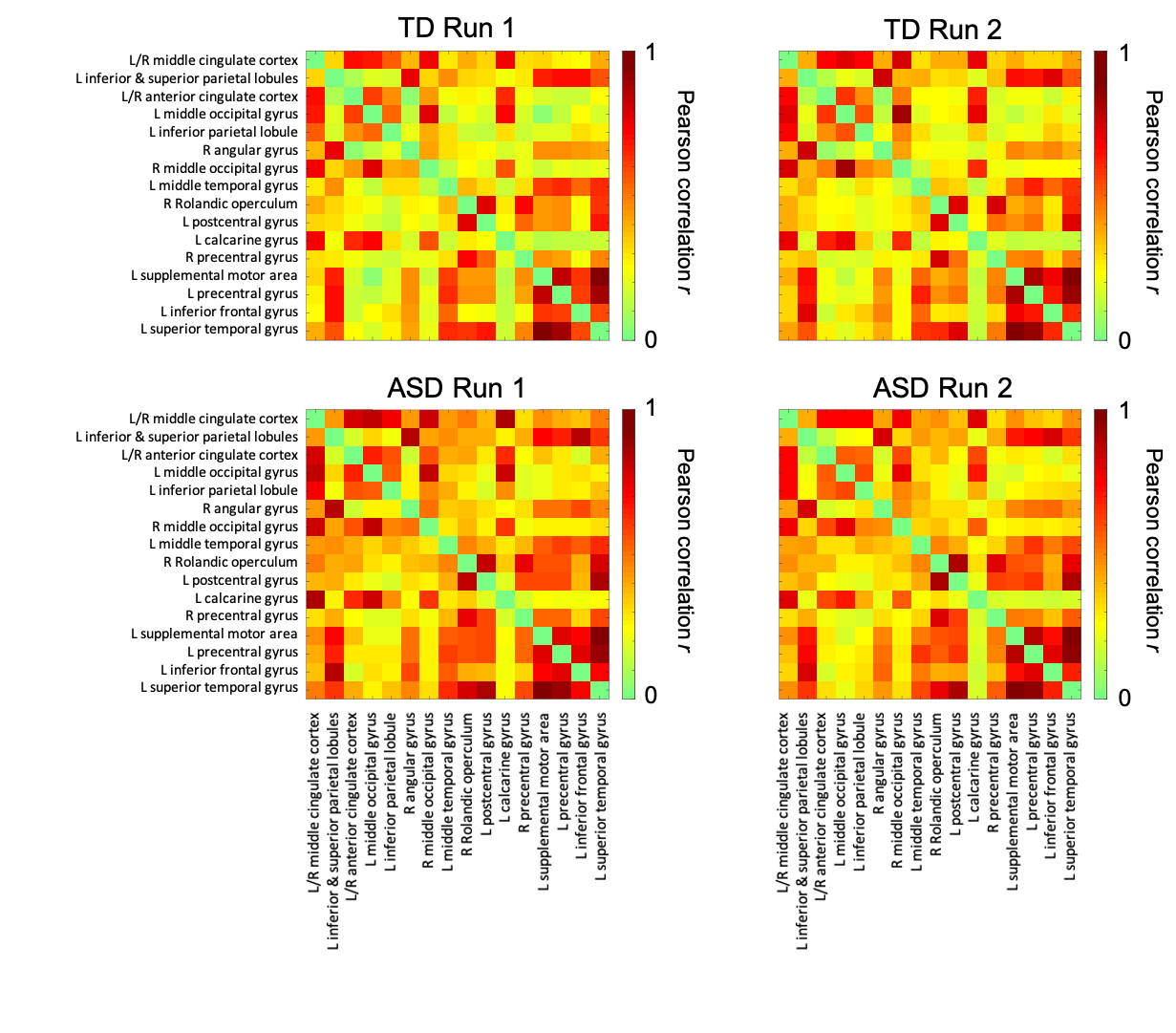


Supplemental Figure S1. Matrix of task FC for Each Task Run


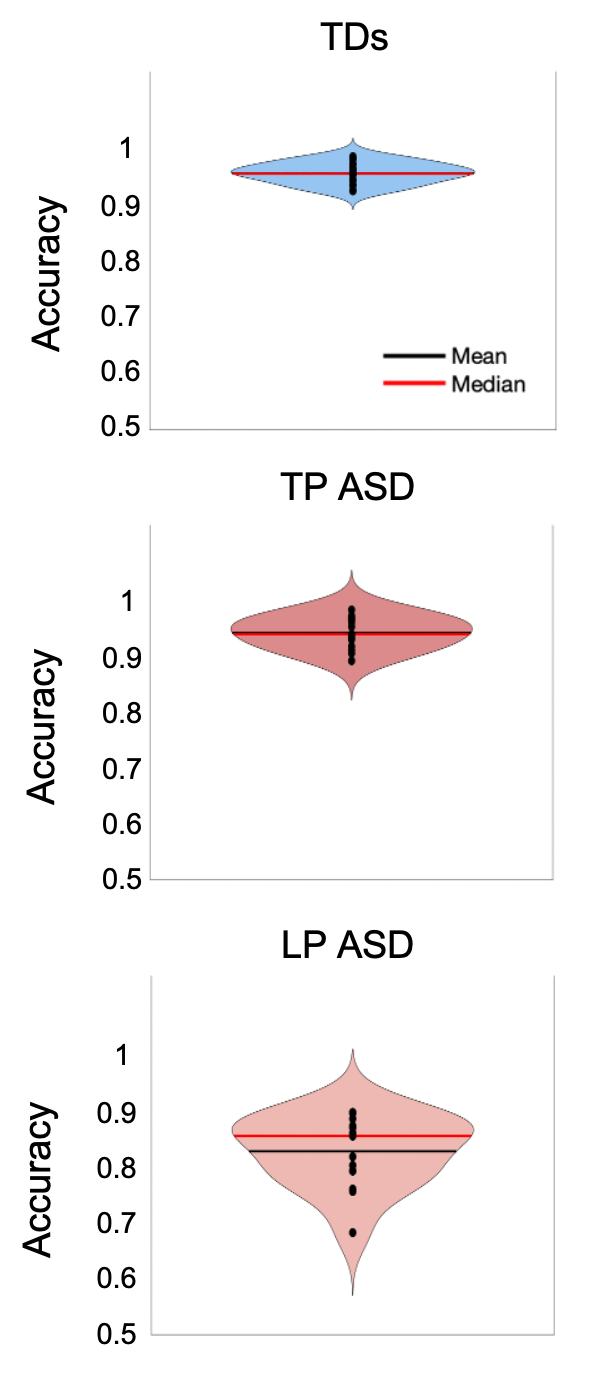


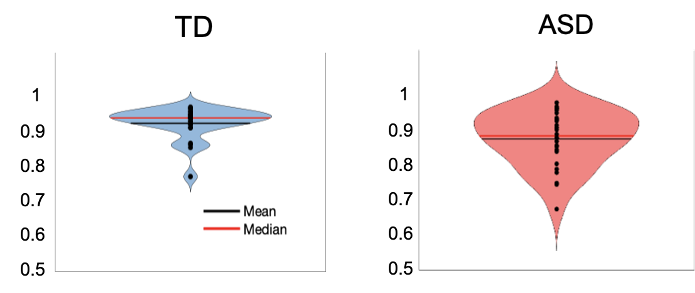


Supplemental Figure S2. Violin Plots of Performance Accuracy in ASD and TD


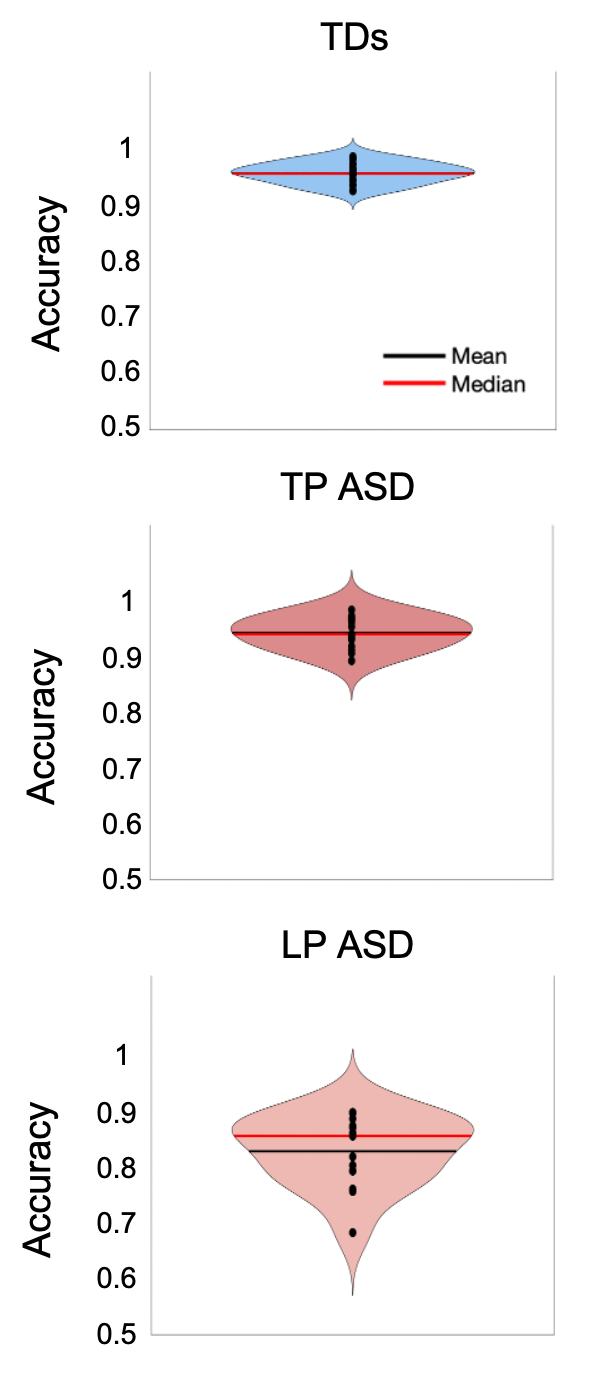


Supplemental Figure S3. Violin Plots of Performance Accuracy in TD_s_, TP-ASD and LP-ASD

**
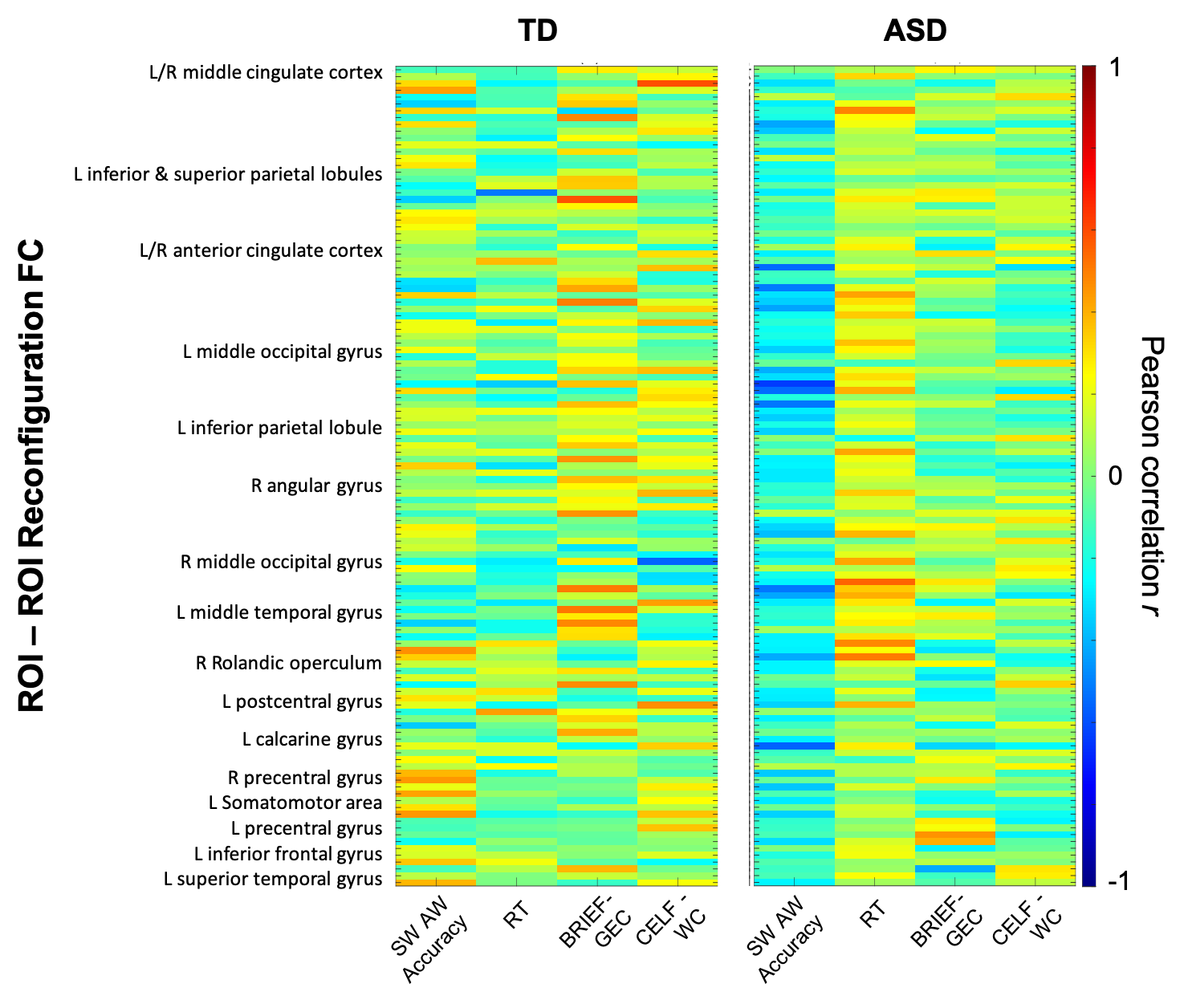
**

**
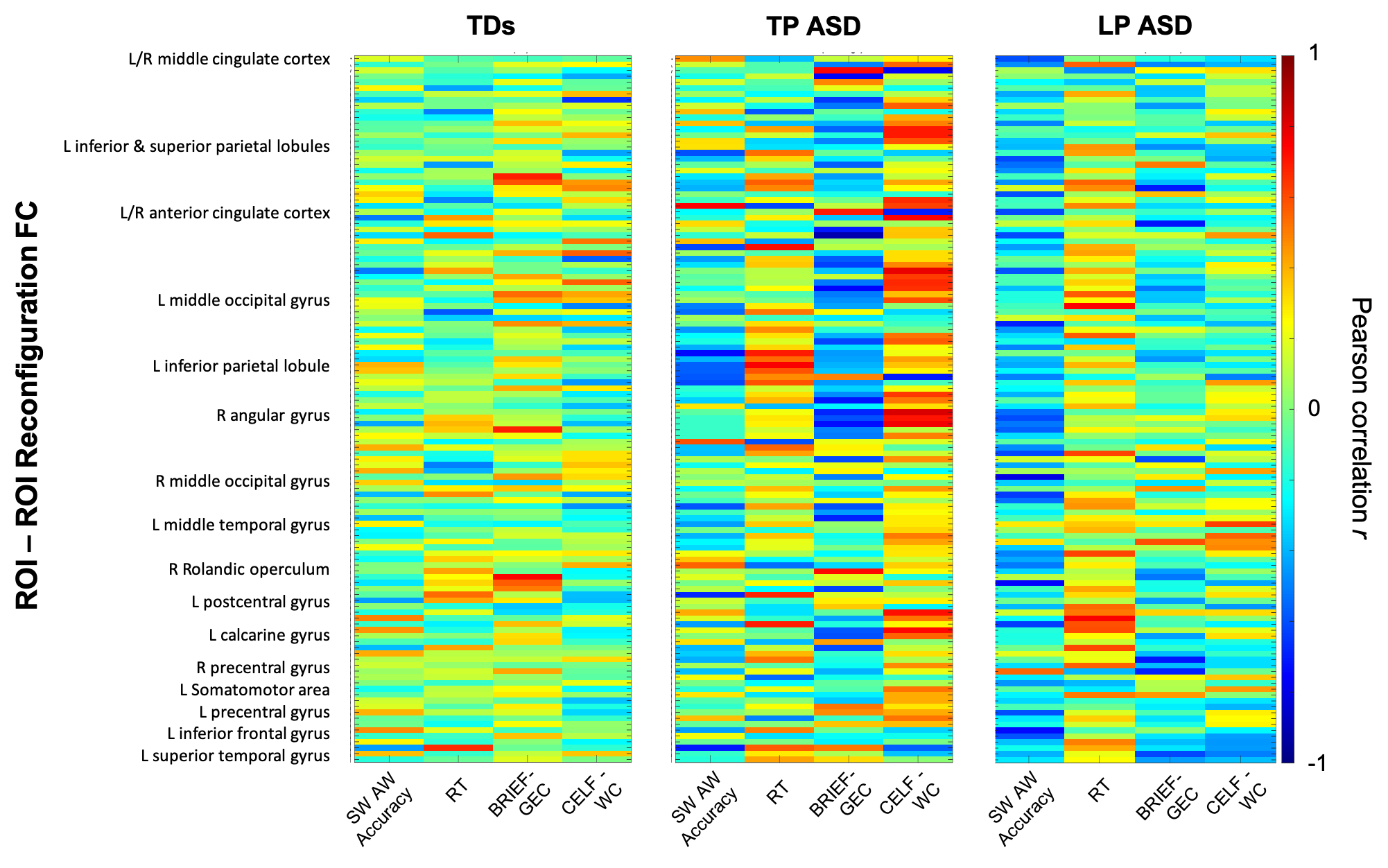
**

Supplemental Figure S4. Heat maps of correlation matrices of FC reconfiguration with behavioral measures ([SW, AW] accuracy, RT, BRIEF-2 GEC scores and CELF-5 WC scores) for ASD and TD (top row); TP-ASD, LP-ASD and TD_s_ (bottom row). Y-axis label shows the common ROI in the ROI-to-ROI pair.
